# Machine Learning–Based Prediction of COVID-19 Mortality With Limited Attributes to Expedite Patient Prognosis and Triage: Retrospective Observational Study

**DOI:** 10.2196/29392

**Published:** 2021-10-15

**Authors:** Riccardo Doyle

**Affiliations:** 1 Stuart Ltd London United Kingdom

**Keywords:** COVID-19, coronavirus, medical informatics, machine learning, artificial intelligence, dimensionality reduction, automation, model development, prediction, hospital, resource management, mortality, prognosis, triage, comorbidities, public data, epidemiology, pre-existing conditions

## Abstract

**Background:**

The onset and development of the COVID-19 pandemic have placed pressure on hospital resources and staff worldwide. The integration of more streamlined predictive modeling in prognosis and triage–related decision-making can partly ease this pressure.

**Objective:**

The objective of this study is to assess the performance impact of dimensionality reduction on COVID-19 mortality prediction models, demonstrating the high impact of a limited number of features to limit the need for complex variable gathering before reaching meaningful risk labelling in clinical settings.

**Methods:**

Standard machine learning classifiers were employed to predict an outcome of either death or recovery using 25 patient-level variables, spanning symptoms, comorbidities, and demographic information, from a geographically diverse sample representing 17 countries. The effects of feature reduction on the data were tested by running classifiers on a high-quality data set of 212 patients with populated entries for all 25 available features. The full data set was compared to two reduced variations with 7 features and 1 feature, respectively, extracted using univariate mutual information and chi-square testing. Classifier performance on each data set was then assessed on the basis of accuracy, sensitivity, specificity, and received operating characteristic–derived area under the curve metrics to quantify benefit or loss from reduction.

**Results:**

The performance of the classifiers on the 212-patient sample resulted in strong mortality detection, with the highest performing model achieving specificity of 90.7% (95% CI 89.1%-92.3%) and sensitivity of 92.0% (95% CI 91.0%-92.9%). Dimensionality reduction provided strong benefits for performance. The baseline accuracy of a random forest classifier increased from 89.2% (95% CI 88.0%-90.4%) to 92.5% (95% CI 91.9%-93.0%) when training on 7 chi-square–extracted features and to 90.8% (95% CI 89.8%-91.7%) when training on 7 mutual information–extracted features. Reduction impact on a separate logistic classifier was mixed; however, when present, losses were marginal compared to the extent of feature reduction, altogether showing that reduction either improves performance or can reduce the variable-sourcing burden at hospital admission with little performance loss. Extreme feature reduction to a single most salient feature, often age, demonstrated large standalone explanatory power, with the best-performing model achieving an accuracy of 81.6% (95% CI 81.1%-82.1%); this demonstrates the relatively marginal improvement that additional variables bring to the tested models.

**Conclusions:**

Predictive statistical models have promising performance in early prediction of death among patients with COVID-19. Strong dimensionality reduction was shown to further improve baseline performance on selected classifiers and only marginally reduce it in others, highlighting the importance of feature reduction in future model construction and the feasibility of deprioritizing large, hard-to-source, and nonessential feature sets in real world settings.

## Introduction

Prior to the COVID-19 pandemic, hospitals in several countries were already experiencing difficulty in managing scarce resources and staff dissatisfaction.

In the United Kingdom, occupancy rates have steadily increased for a decade, with general bed occupancy rising from 84.3% in 2010 to 89.4% in 2019 (92% for general and acute care beds) [1]; meanwhile, overall bed stock across the European Union declined by 2.5% between 2013 and 2018 [2].

In several countries, such as Italy, Greece, and Portugal, this decrease in occupancy rates is set against a financial backdrop of decreasing public health spend, with each listed country registering a decrease in per capita government health care expenditure between 2010 and 2018 [3]. In addition to geographically localized reductions in funding, overall spend has been edging away from acute care and hospital services, with expenditure on inpatient care across Organisation for Economic Co-operation and Development countries growing 14% slower than expenditure on outpatient care and 23% slower than expenditure on long term care between 2013 and 2017 [4].

Staff satisfaction and supply have also proved troublesome. In a cross-sectional US study that was performed shortly prior to the onset of the COVID-19 pandemic, 70% of nurses across hundreds of surveyed institutions stated they would not recommend their hospital; half experienced high burnout, and one-fourth stated they planned to leave the profession within a year [5].

The increased demands imposed by the spread of COVID-19 have in many cases exacerbated the above areas of concern. Lack of resource management protocols and stock limitations led to a shortage of hospital beds [6] and ventilators [7,8] in the early stages of the pandemic, while in some cases, contraction of the virus by medical staff has as much as doubled sickness absence rates [9], further straining staff availability and supply.

These shortcomings have direct adverse consequences for patient care, with a study of 4453 hospitals in the United States from the early phase of the pandemic finding that lower numbers of intensive care unit beds, nurses, and general medicine beds per COVID-19 case were significantly associated with a higher rate of death [10].

To improve use of equipment and better manage physician and nurse supply, increased focus has been brought to information technology. Of particular interest to this paper are computational models that are capable of predicting mortality using real-time patient data. Such models aim to reduce hospital burden by providing efficient patient triage, allowing for preallocation or local hospital transfer of lifesaving equipment, quantifying the need for further diagnostics or early treatment, and directing limited staff attention and resources toward the patients at highest risk.

Several such models have now entered the academic literature, but with varying degrees of usability. Many suffer from mild to severe flaws, such as training on alternative diseases such as pneumonia as a proxy for COVID-19 [11], depending on less immediately available data from blood tests and other monitoring equipment [12-15], and unrepresentative population samples—often older skewing [16] or monolocalized [17]; these flaws result either in low performance or, more worryingly, in excessively optimistic expectations of performance that overfit to a certain facet of the population.

Although all predictive models will inevitably suffer from issues surrounding quality of data or population reproducibility, many of them still generate valuable findings that can materially aid in patient profiling and optimization of treatment and have been adopted on a supportive level by hospitals.

In this study, we aim to further the utility of existing models by exploring the impact of dimensionality reduction on the predictive accuracy of patient outcomes, providing a use case for reduction of costly or slowly available patient attributes, such as laboratory or imaging results, in favor of simple patient history and demographics.

## Methods

### Data Set

Data for this study were obtained from a continuously updated repository [18] containing anonymized patient level information on 2,676,311 COVID-19–positive individuals across 146 countries. The results represent data sourced on February 18, 2021, encompassing entries up to and including the date in question.

### Variable Extraction and Data Preprocessing

Symptoms and comorbidities in the data set were parsed and one-hot encoded into fixed variable names. Textbox 1 shows all the patient variables used in the study.

Of the original 2,676,311 patients, only 212 patients had fully populated fields for all the above variables; these patients were retained in the study.

**Features employed as predictors of mortality by category.**

**Symptoms**

- Cough
- Fever
- Runny nose
- Fatigue
- Headache
- Diarrhea
- Sore throat
- Chest symptoms
- Chills
- Difficulty breathing
- Acute respiratory distress syndrome
- Pneumonia

**Pre-existing conditions**

- Benign prostatic hyperplasia and other prostate conditions
- Coronary heart disease or other cardiac condition
- Chronic kidney disease and other kidney conditions
- Hypertension
- Diabetes
- Pulmonary conditions
- Asthma
- Bronchitis
- Conditions affecting the arteries
- Cancer

**Demographic attributes**

- Age
- Sex

**Derived attributes**

- Number of pre-existing conditions

### Dimensionality Reduction

Dimensionality reduction was applied to compare a full 25-feature data set to 7-feature and 1-feature reduced variations. Feature selection was performed, first through mutual information and second through chi-square tests, to compare the selection impact of different approaches.

Both frameworks were executed in Python using the *mutual_info_classif* and *chi2* methods in scikit-learn’s *feature_selection* module [19]. The only continuous variable in the data set, age, was discretized through bins in the chi-square method and through a *k*-nearest neighbors approach in the mutual information method; a more comprehensive definition of the latter can be found in the relevant cited work [20].

Features were extracted using the extent of *P* value significance in the chi-square framework and mutual information in the alternative method, where the latter can be broadly defined as [21]:



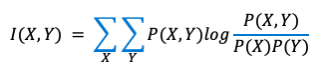



where X is a predictor feature in the data and Y is an outcome of death or recovery.

### Predictive Models and Evaluation Criteria

Random forest and logistic regression were employed as primary classifiers to be trained on ex ante balanced data and tested on unprocessed imbalanced data.

Model performance was evaluated based on accuracy, area under the curve (AUC), sensitivity, and specificity. Sensitivity measures the proportion of deaths correctly identified by the model, expressed as:



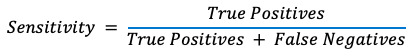



Where death is a positive outcome and recovery is a negative outcome. Specificity measures the proportion of recoveries correctly identified, expressed as:



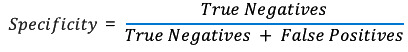



All metrics were derived from aggregation during 3-fold cross-validation.

## Results

### Sample Baseline Characteristics

The fully populated data set contains full entries for each category mentioned in the methodology, resulting in a sample of 212 patients. The data are geographically diverse, with representation from 17 countries, although 62/212 patients (29.2%) originate from China alone. The mean age in the sample is 55.9 (SD 21.8) years. The mean age of patients who died of COVID-19 is significantly higher than that of those who did not, at 64.1 (SD 19.6) years against 40.8 (SD 16.9) years, respectively. Men comprised 67.9% (144/212) of the sample. A sizeable 49.5% (105/212) of the sample suffered from a pre-existing condition, which is overrepresented, and 64.6% (137/212) of the sample ultimately died, rendering the final class balance highly skewed.

### Correlation Matrix of Features

**Figure 1:**
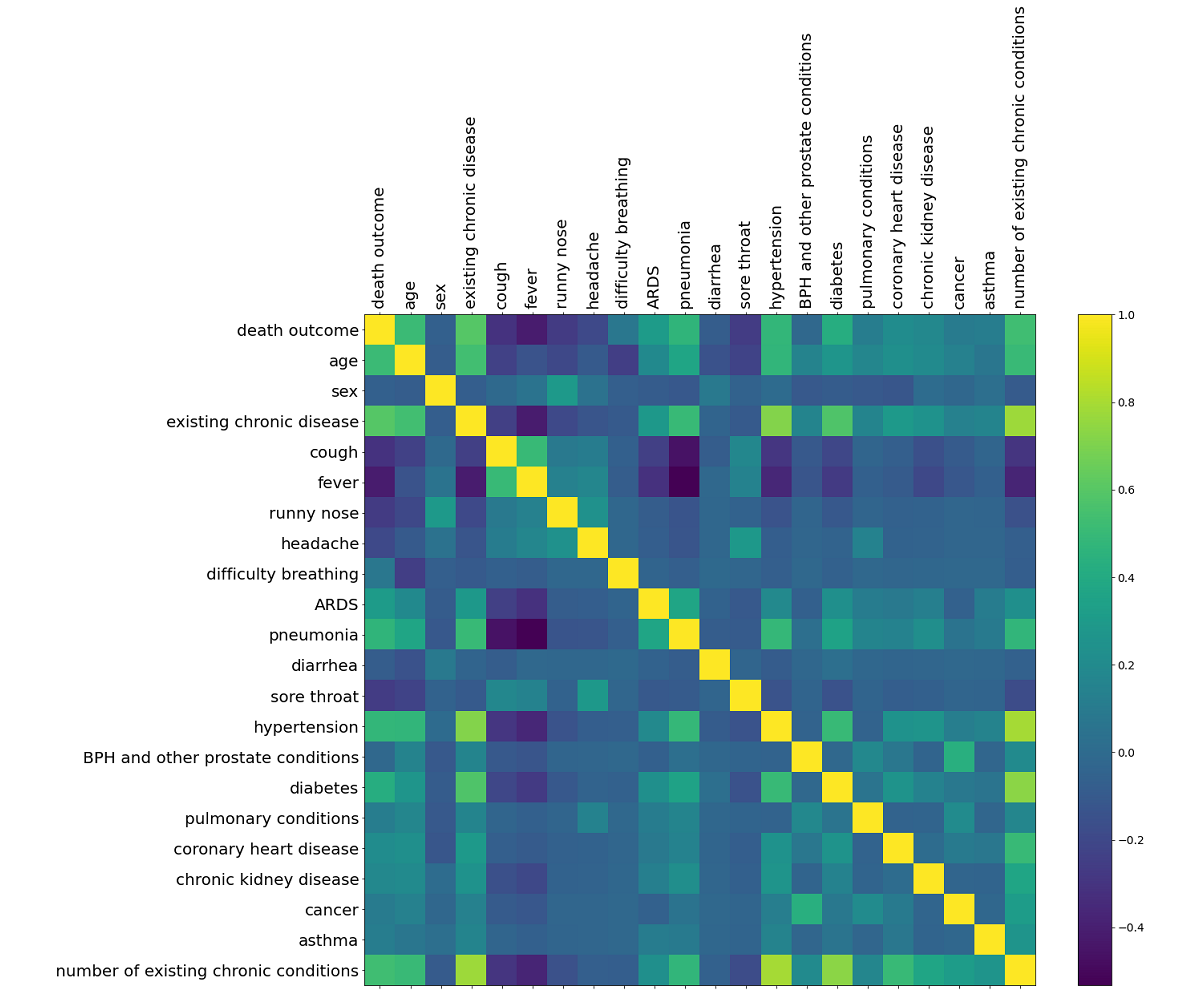
Correlation matrix of patient demographics, symptoms, and pre-existing conditions with each other and with an outcome of death. ARDS: acute respiratory distress syndrome; BPH: benign prostatic hyperplasia.

Before analyzing the prediction model performance, Figure 1 outlines the main cross-correlation of patient characteristics and their correlation with an outcome of death. We note that the most explanatory features raising mortality risk are age (correlation coefficient 0.51), whether a patient has a pre-existing chronic condition (correlation coefficient 0.59), and the number of pre-existing conditions (correlation coefficient 0.53). This is followed by particularly risk-elevating conditions, such as diabetes and hypertension, and specific symptoms of advanced disease progression, such as pneumonia and acute respiratory distress syndrome.

### Feature Importance Analysis

As outlined in the methodology, dimensionality reduction techniques were applied to generate two extracted data sets, one with 7 features and one with 1 feature. The extraction was repeated at each cross-validation fold to avoid lookahead bias, making the final feature sets less auditable. In this section, we anticipate subsequent results by providing a brief overview of the 7 most salient features (Table 1) selected solely using mutual information across the entire 212-patient sample rather than individual training folds.

We note that with the exception of fever, symptoms were not featured in the reduction, in favor of the increased importance of pre-existing conditions and age as a general all-encompassing feature. Among comorbidities, diabetes and hypertension stood ahead of the others in impact, while the overall number of concomitant comorbidities in a single patient was also significant.

**Table 1:** Features included in a reduced 7-variable data set derived using mutual information on the full 212-patient data set.

Feature	Univariate mutual information
Age	0.35
Number of chronic diseases	0.22
Presence of chronic diseases	0.20
Hypertension	0.19
Pneumonia	0.11
Fever	0.09
Diabetes	0.09

### Mutual Information Feature Reduction

This study compares the impact of two dimensionality reduction methods—mutual information and chi-square tests—on model performance. Table 2 outlines the out-of-sample performance of models trained on the full 25-feature data set compared to 7-feature and 1-feature variations extracted through mutual information. All metrics were calculated over multiple repetitions of 3-fold cross validation. Due to differences in class balance across folds and simulated repetitions, accuracy metrics are not necessarily a weighted average of their sensitivity and specificity.

We note that performance across all models and datasets is sound, with no accuracy below 79.2%. The best random forest classifier performed substantially above the best logistic classifier, with respective accuracies of 90.8% and 83.5%.

In assessing the impact of dimensionality reduction, we note that transitioning from 25 features to 7 improved the performance of the random forest classifier (89.2% to 90.8% accuracy), while a minor (considering the extent of feature shrinkage) reduction in performance was observed in the logistic classifier (83.5% to 79.2%). The latter may be overstated by the model’s decision threshold, as the AUC decrease was minor (88.6% to 88.4%).

Finally, extreme reduction to a single most important attribute—in most folds, age—resulted in substantially reduced performance in the random forest classifier (89.2% to 80.1% compared to the full data set baseline) but more muted loss in the logistic classifier (83.5% to 81.5%).

All models were drawn from Python’s scikit-learn libraries. For reproducibility, random forest models were run with 100 estimator trees and a 2-sample minimum split criterion, while logistic models were run on default parameters with no regularization.

**Table 2:** Mortality prediction performance of selected classifiers on various reduced data sets extracted via mutual information.

Model and data set granularity			Average values across folds (%)						
			Specificity^a^ (95% CI)		Sensitivity^a^ (95% CI)		Accuracy^a^ (95% CI)		AUC^b^ (95% CI)
**Random forest**									
	25-feature data set	83.2 (80.1-86.3)		89.1 (86.8-91.4)		89.2 (88.0-90.4)		96.3 (95.9-96.6)	
	7-feature data set	88.3 (86.2-90.6)		90.0 (88.3-91.6)		90.8 (89.8-91.7)		95.0 (94.4-95.5)	
	1-feature data set	84.9 (83.3-86.2)		76.8 (74.9-78.7)		80.1 (79.8-81.7)		88.1 (87.1-89.0)	
**Logistic regression**									
	25-feature data set	82.9 (79.9-85.9)		79.6 (76.0-83.2)		83.5 (81.9-85.1)		88.6 (87.5-89.7)	
	7-feature data set	86.5 (83.9-89.1)		70.3 (65.4-75.2)		79.2 (76.9-81.5)		88.4 (87.4-89.4)	
	1-feature data set	80.3 (79.4-81.3)		80.7 (79.3-82.0)		81.5 (81.0-82.0)		84.2 (83.4-84.9)	

^a^Reported performance metrics represent averages across multiple simulations of 3-fold cross validation and, due to class balance variation between folds, accuracy metrics are not always a weighted average of their sensitivity and specificity.

^b^AUC: area under the curve obtained from the receiver operating characteristic curve.

### Chi-Square Feature Reduction

The impact of mutual information having been assessed, Table 3 outlines the out-of-sample performance of models training on reduced data sets extracted via chi-square significance rather than mutual information.

We note that the trends are generally similar between reduction methods, but some important divergences are present. Random forest performance improvement in transitioning from 25 features to 7 is larger when extracting features through chi-square significance, with accuracy now improving from 89.2% to 92.5%. Performance loss in the logistic classifier is also less severe, falling from 83.5% to only 79.8% while the AUC increases (from 88.6% to 89.5%).

Performance differentials in transitioning to a single feature data set are similar to those summarized using mutual information.

**Table 3:** Mortality prediction performance of selected classifiers on various reduced data sets extracted via chi-square significance.

Model and data set granularity			Average values across folds (%)					
			Specificity^a^ (95% CI)		Sensitivity^a^ (95% CI)		Accuracy^a^ (95% CI)	AUC^b^ (95% CI)
**Random forest**								
	25-feature data set	83.2 (80.1-86.3)		89.1 (86.8-91.4)		89.2 (88.0-90.4)		96.3 (95.9-96.6)
	7-feature data set	90.7 (89.1-92.3)		92.0 (91.0-92.9)		92.5 (91.9-93.0)		95.5 (95.2-95.8)
	1-feature data set	84.8 (83.3-86.2)		77.6 (76.6-78.6)		81.1 (80.7-81.6)		88.5 (88.1-89.0)
**Logistic regression**								
	25-feature data set	82.9 (79.9-85.9)		79.6 (76.0-83.2)		83.5 (81.9-85.1)		88.6 (87.5-89.7)
	7-feature data set	90.4 (89.1-91.8)		69.9 (64.9-74.8)		79.8 (77.3-82.1)		89.5 (88.4-90.6)
	1-feature data set	80.2 (79.4-81.0)		80.9 (79.8-82.1)		81.6 (81.1-82.1)		84.2 (83.5-84.9)

^a^Reported performance metrics represent averages across multiple simulations of 3-fold cross validation and, due to class balance variation between folds, accuracy metrics are not always a weighted average of their sensitivity and specificity.

^b^AUC: area under the curve obtained from the receiver operating characteristic curve.

## Discussion

### Principal Findings

Models trained on a high-quality 212-patient data set containing 25 symptom, comorbidity, and demographic variables showed strong detection ability, with the highest-performing model achieving specificity of 90.7% (95% CI 89.1%-92.3%) and sensitivity of 92.0% (95% CI 91.0%-92.9%). The impact of dimensionality reduction on performance was explored by extracting features, first via mutual information and second via chi-square significance, to create two reduced data sets, one containing 7 features and one containing a single feature.

Application of either mutual information or chi-square significance to reduce the data set to 7 features resulted in improvement of the predictive performance when using a random forest classifier and in mixed performance variation when using a logistic classifier. These results strongly suggest that dimensionality reduction can be beneficial to model performance or can provide reduced dependence on large feature sets at minimal cost to performance. We also note that all models tested on either the 25-feature or 7-feature data sets performed roughly in line with or not much worse than existing studies drawing on a plethora of additional blood markers and vitals [12-15].

Further analyzing the effect of dimensionality reduction between reduction methods showed disparity in the final performance impact. Comparing the performance of a random forest classifier trained on 25 features against that trained on 7 features derived through feature extraction, mutual information reduction resulted in an increase in accuracy from 89.2% to 90.8%, compared to 92.5% when employing chi-square methods. In the logistic model, mutual information resulted in a decrease in accuracy from 83.5% to 79.2%, compared to 79.8% using chi-square methods; additionally, it should be noted that although the AUC decreased from 88.6% to 88.4% in the former case, it substantially increased to 89.5% in the latter. This suggests there can be significant variation in performance based on the choice of reduction methods, and it is strongly advised that in future studies—especially those containing hundreds of features initially sifted by feature extraction methods—a wide array of dimensionality reduction methods should be employed and tested as hyperparameters when cross-validating models as opposed to arbitrarily selecting one ex ante.

Beyond reduction to 7 features, an extreme reduction to a single most salient feature—often age—was tested to highlight the ability of the models to generalize in even the most constraining scenario; this resulted in decreased but still sound predictive performance and demonstrated the high baseline predictive power that age or other salient comorbidity variables have as standalone variables in mortality detection, with remaining variables providing marginal additional explanatory power.

Variable importance using mutual information was explored by reporting the 7 most salient features; symptom data—with the exception of fever—were found to be less impactful than comorbidities (particularly hypertension and diabetes), age, and a proxy for the number of concomitant comorbidities. Studies [22] with a similar focus on feature extraction confirm the importance of these same selected features, with additions such as chronic obstructive pulmonary disease and heart failure, although variations in importance can be noted by age strata.

The classifiers explored in this study have been shown to have very high mortality prediction accuracy, confirming the utility of this class of models for patient prognosis and triage. In hospital settings, patient histories at admission include all relevant attributes necessary to obtain a mortality prediction from our classifiers, resulting in timely assignment to relevant wards based on risk level, allocation of additional monitoring equipment, consideration for additional screenings, escalation to a secondary prediction model with more elaborate features, or allocation of scarce or experimental preventive treatment.

### Limitations

The study’s main limitations are the period its data relate to, which spans the first 4 months of the COVID-19 pandemic and does not include information on new variants of concern or current dominant strains, and the relatively even class balance in the sample, with 64.6% of the 212 patients (n=137) dying from disease progression. The latter in particular means that the performance reported above, while representative, could experience a greater disparity in sensitivity and specificity balance when testing on the more uneven class balance implied by the current COVID-19 mortality rate.

### Conclusion

This study has confirmed the substantial accuracy that machine learning models can bring to the early detection of mortality in COVID-19. Additionally, we demonstrated that dimensionality reduction can at best further increase said accuracy or at worst materially aid hospitals in reducing the number of diagnostic variables needed before obtaining usable predictions with only marginal costs to performance.

## Abbreviations

AUC: area under the curve

## References

[1] (2021). Bed availability and occupancy data – overnight. National Health Service.

[2] (2020). Healthcare resource statistics - beds. Eurostat.

[3] Prante FJ, Bramucci A, Truger A (2020). Decades of tight fiscal policy have left the health care system in Italy ill-prepared to fight the COVID-19 outbreak. Inter Econ.

[4] (2019). Health at a Glance. Organisation for Economic Co-operation and Development.

[5] Lasater KB, Aiken LH, Sloane DM, French R, Martin B, Reneau K, Alexander M, McHugh MD (2021). Chronic hospital nurse understaffing meets COVID-19: an observational study. BMJ Qual Saf.

[6] Mateen BA, Wilde H, Dennis JM, Duncan A, Thomas N, McGovern A, Denaxas S, Keeling M, Vollmer S (2021). Hospital bed capacity and usage across secondary healthcare providers in England during the first wave of the COVID-19 pandemic: a descriptive analysis. BMJ Open.

[7] Iyengar K, Bahl S, Vaish A, Raju Vaishya (2020). Challenges and solutions in meeting up the urgent requirement of ventilators for COVID-19 patients. Diabetes Metab Syndr.

[8] Ranney ML, Griffeth V, Jha AK (2020). Critical supply shortages — the need for ventilators and personal protective equipment during the Covid-19 pandemic. N Engl J Med.

[9] Appleby J (2021). NHS sickness absence during the covid-19 pandemic. BMJ.

[10] Janke AT, Mei H, Rothenberg C, Becher RD, Lin Z, Venkatesh AK (2021). Analysis of hospital resource availability and COVID-19 mortality across the United States. J Hosp Med.

[11] Barda N, Riesel D, Akriv A, Levy J, Finkel U, Yona G, Greenfeld D, Sheiba S, Somer J, Bachmat E, Rothblum GN, Shalit U, Netzer D, Balicer R, Dagan N (2020). Developing a COVID-19 mortality risk prediction model when individual-level data are not available. Nat Commun.

[12] El-Solh AA, Lawson Y, Carter M, El-Solh DA, Mergenhagen KA (2020). Comparison of in-hospital mortality risk prediction models from COVID-19. PLoS One.

[13] Knight Stephen R, Ho Antonia, Pius Riinu, Buchan Iain, Carson Gail, Drake Thomas M, Dunning Jake, Fairfield Cameron J, Gamble Carrol, Green Christopher A, Gupta Rishi, Halpin Sophie, Hardwick Hayley E, Holden Karl A, Horby Peter W, Jackson Clare, Mclean Kenneth A, Merson Laura, Nguyen-Van-Tam Jonathan S, Norman Lisa, Noursadeghi Mahdad, Olliaro Piero L, Pritchard Mark G, Russell Clark D, Shaw Catherine A, Sheikh Aziz, Solomon Tom, Sudlow Cathie, Swann Olivia V, Turtle Lance Cw, Openshaw Peter Jm, Baillie J Kenneth, Semple Malcolm G, Docherty Annemarie B, Harrison Ewen M, ISARIC4C investigators (2020). Risk stratification of patients admitted to hospital with covid-19 using the ISARIC WHO Clinical Characterisation Protocol: development and validation of the 4C Mortality Score. BMJ.

[14] Vaid A, Somani S, Russak AJ, De Freitas JK, Chaudhry FF, Paranjpe I, Johnson KW, Lee SJ, Miotto R, Richter F, Zhao S, Beckmann ND, Naik N, Kia A, Timsina P, Lala A, Paranjpe M, Golden E, Danieletto M, Singh M, Meyer D, O'Reilly PF, Huckins L, Kovatch P, Finkelstein J, Freeman RM, Argulian E, Kasarskis A, Percha B, Aberg JA, Bagiella E, Horowitz CR, Murphy B, Nestler EJ, Schadt EE, Cho JH, Cordon-Cardo C, Fuster V, Charney DS, Reich DL, Bottinger EP, Levin MA, Narula J, Fayad ZA, Just AC, Charney AW, Nadkarni GN, Glicksberg BS (2020). Machine learning to predict mortality and critical events in a cohort of patients with COVID-19 in New York City: model development and validation. J Med Internet Res.

[15] Chung H, Ko H, Kang WS, Kim KW, Lee H, Park C, Song H, Choi T, Seo JH, Lee J (2021). Prediction and feature importance analysis for severity of COVID-19 in South Korea using artificial intelligence: model development and validation. J Med Internet Res.

[16] Aktar S, Ahamad MM, Rashed-Al-Mahfuz M, Azad A, Uddin S, Kamal A, Alyami SA, Lin P, Islam SMS, Quinn JM, Eapen V, Moni MA (2021). Machine learning approach to predicting COVID-19 disease severity based on clinical blood test data: statistical analysis and model development. JMIR Med Inform.

[17] An C, Lim H, Kim D, Chang JH, Choi YJ, Kim SW (2020). Machine learning prediction for mortality of patients diagnosed with COVID-19: a nationwide Korean cohort study. Sci Rep.

[18] Xu B, Gutierrez B, Mekaru S, Sewalk K, Goodwin L, Loskill A, Cohn EL, Hswen Y, Hill SC, Cobo MM, Zarebski AE, Li S, Wu C, Hulland E, Morgan JD, Wang L, O'Brien Katelynn, Scarpino S, Brownstein JS, Pybus OG, Pigott DM, Kraemer MUG (2020). Epidemiological data from the COVID-19 outbreak, real-time case information. Sci Data.

[19] Feature selection. scikit-learn.

[20] Ross BC (2014). Mutual information between discrete and continuous data sets. PLoS One.

[21] Kraskov A, Stögbauer H, Grassberger P (2004). Estimating mutual information. Phys Rev E.

[22] Estiri H, Strasser ZH, Klann JG, Naseri P, Wagholikar KB, Murphy SN (2021). Predicting COVID-19 mortality with electronic medical records. NPJ Digit Med.

